# Influence of Catechol-O-Methyltransferase Gene Polymorphism on the Correlation between Alexithymia and Hypervigilance to Pain

**DOI:** 10.3390/ijerph182413265

**Published:** 2021-12-16

**Authors:** Hitomi Ikarashi, Naofumi Otsuru, Hirotake Yokota, Kazuaki Nagasaka, Kazuki Igarashi, Shota Miyaguchi, Hideaki Onishi

**Affiliations:** 1 Graduate School, Niigata University of Health and Welfare, Niigata 950-3198, Japan; hpm20001@nuhw.ac.jp (H.I.); yokota@nuhw.ac.jp (H.Y.); onishi@nuhw.ac.jp (H.O.); 2 Institute for Human Movement and Medical Sciences, Niigata University of Health and Welfare, Niigata 950-3198, Japan; nagasaka@nuhw.ac.jp (K.N.); miyaguchi@nuhw.ac.jp (S.M.); 3 Department of Physical Therapy, Faculty of Rehabilitation, Niigata University of Health and Welfare, Niigata 950-3198, Japan; hpa17005@nuhw.ac.jp

**Keywords:** catechol-O-methyltransferase gene polymorphism, alexithymia, hypervigilance to pain, 20-item Toronto Alexithymia Scale, pain vigilance and awareness questionnaire

## Abstract

The psychological characteristic of having difficulty expressing emotions, known as alexithymia, is associated with hypervigilance to pain and is considered one of the risk factors for chronic pain. The correlation between alexithymia and hypervigilance to pain can be observed even in healthy individuals. However, the factors influencing this correlation remain unknown. We explored the dopamine system, which is known to be involved in emotion and pain. The dopamine-degrading enzyme catechol-O-methyltransferase (COMT) has a genetic polymorphism known to influence dopamine metabolism in the prefrontal cortex. COMT polymorphism reportedly affects various aspects of pain and increases pain sensitivity in Met allele carriers. Therefore, we investigated whether the correlation between alexithymia and hypervigilance to pain is influenced by COMT polymorphism in healthy individuals. The results revealed a significant positive correlation between the “difficulty describing feelings” of the 20-item Toronto Alexithymia Scale and the “attention to changes in pain” of the pain vigilance and awareness questionnaire in COMT Met carriers but not in Val/Val individuals. This finding suggests that the correlation between alexithymia and hypervigilance to pain is influenced by COMT polymorphism.

## 1. Introduction

Alexithymia—a psychological trait of patients with psychosomatic conditions—was first described by Sifneos, and it refers to difficulty in expressing one’s emotions [1]. Although the prevalence of alexithymia is >10% in the general population, it is substantially higher in patients with psychosomatic disorders (40–60%) [2,3]. Alexithymia is assessed using the 20-item Toronto Alexithymia Scale (TAS-20) [4], which comprises three subscales: difficulty identifying feelings (DIF), difficulty describing feelings (DDF), and externally oriented thinking (EOT).

Previous studies have shown that high TAS-20 scores are associated with a high sensitivity to the stimulation of the body (i.e., somatosensory amplification) [5,6,7]. A higher TAS-20 score is associated with an increased risk of chronic pain; moreover, the TAS-20 score positively correlates with pain intensity in patients with chronic pain [8,9]. The TAS-20 scores have been reported to correlate with hypervigilance to pain, as assessed by the pain vigilance and awareness questionnaire (PVAQ) [10]. Taken together, these studies indicate a close association of alexithymia with pain state. A higher TAS-20 score is also associated with hypersensitivity to experimental pain, even in healthy individuals [5,11,12]. This suggests that a correlation between alexithymia and hypersensitivity to pain exists not only in patients with chronic pain but also in the general population. However, the neural underpinnings of the correlation between alexithymia and pain remain unclear.

In the present study, we focused on dopamine function in the brain. Catechol-O-methyltransferase (COMT) is a major enzyme responsible for catecholamine catabolism [13]. A genetic polymorphism has been identified in COMT—Val158Met, in which valine (Val) is replaced by methionine (Met) at codon 158. The Met allele has lower enzyme activity and reduces dopamine resolution. In Val/Val homozygotes, three- to four-fold higher enzyme activity is observed compared with Met/Met homozygotes [14]. The difference in enzyme activity due to the COMT polymorphism is known to modulate dopamine function in the prefrontal cortex [15].

Dopamine function is associated with affective disorders, including alexithymia [16,17]. Previous studies on obsessive compulsive disorder (OCD) have reported that patients with the Val/Val genotype have higher TAS-20 scores than those with the Val/Met or Met/Met genotype [18].

COMT polymorphism also relates to pain perception. A previous study showed that various genetic factors can modulate the perceptual intensity of pain, sensitivity to painful stimuli, and development of chronic pain [19]. Among such genetic factors, COMT is associated with chronic pain conditions [20]. Differences in dopamine function due to COMT polymorphism are considered to be involved in emotional and cognitive dysfunction in patients with chronic pain [21]. Notably, among patients with fibromyalgia, those with the Met/Met genotype are more sensitive to pain than those with the Val/Val genotype [22]. Even among healthy individuals, Met carriers experience more pain than those with the Val/Val genotype [23].

As mentioned above, both alexithymia and pain are involved in dopamine function and are closely related to each other. However, the effect of dopamine function on the strength of the association between alexithymia and pain remains unclear. Therefore, this study investigated whether COMT polymorphism influences the correlation between alexithymia and pain, assessed by the TAS-20 and PVAQ, respectively.

## 2. Materials and Methods

### 2.1. Participants

We recruited 80 healthy individuals (45 men and 35 women; mean age 21 ± 0.5 years) from Niigata University of Health and Welfare, Japan. Individual COMT polymorphism was identified using allelic discrimination real-time polymerase chain reaction (PCR) before the experiment. Genotyping identified 48 participants with the Val/Val genotype (29 men and 19 women), 25 with the Val/Met genotype (11 men and 14 women), and 7 with the Met/Met genotype (4 men and 3 women). To match the groups in terms of number of participants and sex, a total of 36 participants were randomly selected, including men and women with each genotype, from the 80 participants. Thus, we recruited 18 participants (9 men and 9 women) with the Val/Val genotype and 18 participants that were Met carriers (9 men and 9 women: 13 with the Val/Met genotype and 5 with the Met/Met genotype). None of the participants were taking any type of medications and had no neurological or psychiatric disorders. None of these subjects reported any pain on the day they were called to answer the questionnaire. This study was conducted in accordance with the Declaration of Helsinki and was approved by the institutional review board of Niigata University of Health and Welfare (approval number: 18154). Written informed consent was obtained from all participants.

### 2.2. PVAQ

The PVAQ is a measure of hypervigilance to pain. It comprises 16 items (e.g., “I am very sensitive to pain” and “I am quick to notice changes in pain intensity”) that are rated on a 6-point scale from 0 (never) to 5 (always) [24]. It comprises two subscales that measure the attention to pain (the PVAQ-AP; scores 0–50) and attention to changes in pain (the PVAQ-ACP; scores 0–30). Participants completed the Japanese version of the PVAQ, which has been shown to have good internal consistency (Cronbach’s α of 0.89 for the PVAQ-AP and 0.81 for the PVAQ-ACP) [25]. A previous study has reported the cut-off value for the PVAQ (in total) to be ≥24.5 [26].

### 2.3. TAS-20

Alexithymia was assessed in each participant using the TAS-20, which is the most psychometrically valid measurement for alexithymia [4]. The TAS-20 comprises 20 items that are rated on a 5-point scale from 1 (never) to 5 (always). It comprises three subscales: the TAS-20-DIF (scores 0–35), the TAS-20-DDF (scores 0–25), and the TAS-20-EOT (scores 0–40). Participants completed the Japanese version of the TAS-20, which has been shown to have good internal consistency (Cronbach’s α of 0.85, 0.72, and 0.58 for the TAS-20-DIF, TAS-20-DDF, and TAS-20-EOT, respectively) [27]. A previous study has reported the cut-off value for the TAS-20 (in total) to be ≥61 [28].

### 2.4. DNA Amplification and Genotyping of COMT Polymorphism

The sequences for the design of the genotyping assay were obtained from the single nucleotide polymorphism database (COMT-rs4680) of the National Center for Biotechnology Information. The DNA was extracted from whole blood samples using the NucleoSpin Blood Quickpure Kit (Macherey-Nagel, Düren, Germany). The samples were genotyped using TaqMan allelic discrimination real-time PCR using CFX Connect (Bio-Rad Laboratories, CA, USA). The reactions were performed in duplicate using the Kapa Probe Fast qPCR Kit Master Mix (2X) Universal (Kapa Biosystems, Wilmington, MA, USA). We used the following forward and reverse primers, respectively: 5′-GCGGATGGTGGATTTCGC-3′ and 5′-TGACAACGGGTCAGGCA-3′. The probe sequences were FAM-TGGCGTGAAGGACAAGGTGTG-BHQ for probe G and HEX-TGGCATGAAGGACAAGGTGTGC-BHQ for probe A. The primers and probes were synthesized by Nihon Gene Research Laboratories (Miyagi, Japan). The PCR reaction was performed using a 20 µL reaction mixture containing 10 µL of PCR Master Mix, 1 µL of each of the two primers and probes, and 6 µL of DNA and DNase-free water. The amplification was performed using CTX Connect under the following conditions: 95°C for 3 min, followed by 40 cycles at 95 °C for 3 s, 66 °C for 20 s, and 72 °C for 1 s. We determined the fluorescent signal from the HEX- or FAM-labeled probes for each cycle. The discrimination of genotypes was performed using the Bio-Rad CFX manager 3.1 software.

### 2.5. Statistical Analysis

The Shapiro–Wilk test was used to examine the normality of the data. To assess the possible differences in each of the PVAQ and TAS-20 subscale scores between the Val/Val and Met-carrier groups, we used an unpaired *t*- or Mann–Whitney U test. To examine the correlation between the PVAQ and TAS-20 subscales, we used Pearson’s or Spearman’s correlation analyses. For all statistical analyses, *p* < 0.05 was considered significant.

## 3. Results

The results of the Shapiro–Wilk test revealed that the TAS-20-DIF (*p* = 0.036) was non-normally distributed; all other scores were normally distributed. The unpaired *t*- and Mann–Whitney U test results showed no significant differences between the Val/Val and Met-carrier groups in any of the TAS-20 and PVAQ subscales (Table 1). However, the Val/Val group showed a tendency to have higher DIF scores than the Met-carrier group (*p* = 0.059).

The results of the Spearman’s correlation analysis of all participants showed a significant positive correlation between the PVAQ-ACP and TAS-20-DIF scores (r = 0.47, *p* = 0.004). Interestingly, the analyses of each group revealed that this correlation was only significant for the Met-carrier group (r = 0.73, *p* = 0.001) and not the Val/Val group. No significant correlations were observed for any of the other subscales in either group (Table 2).

## 4. Discussion

In the present study, we investigated the effect of COMT polymorphism on the scores of each subscale of the PVAQ and TAS-20 and the correlation between the subscales in healthy individuals. We found no significant differences in any of the PVAQ and TAS-20 subscale scores between the COMT polymorphism groups. This indicated that COMT polymorphism does not affect the psychometric properties of hypervigilance to pain and alexithymia. However, in terms of the correlation between the PVAQ and TAS-20 scores, we found a significant positive correlation between the PVAQ-ACP and TAS-20-DIF scores in the Met-carrier group (but not in the Val/Val group), which suggests that the association between hypervigilance to pain and difficulty in identifying feelings is modulated by COMT polymorphism.

We noted no differences in the TAS-20 and PVAQ scores based on COMT polymorphism, which was consistent with the findings of a previous meta-analysis that also showed that the TAS-20 scores remained unaffected by COMT polymorphism [29]. Moreover, another previous study has reported no difference in the PVAQ scores based on COMT polymorphism [30]. In contrast, some previous studies have reported a significant difference in TAS20 scores based on COMT polymorphism. For example, patients with OCD with the Val/Val genotype have higher total TAS-20, DIF, and DDF scores than Met carriers [18]. Among healthy individuals, those with the Val/Val genotype have been reported to have higher TAS-20 scores than Met carriers [31]. In the present study, we observed that the tendency for having a higher DIF score was higher in participants with the Val/Val genotype than in Met carriers (*p* = 0.059). The relative sample sizes might have influenced this result.

**Figure 1:**
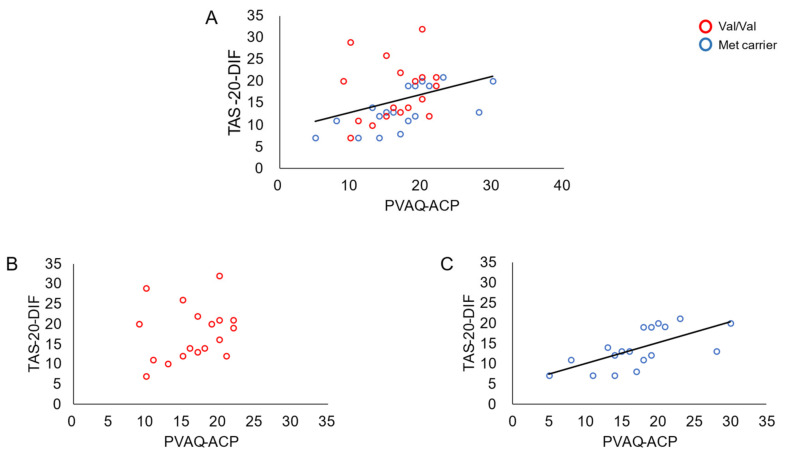
Correlation between the scores of the 20-item Toronto Alexithymia Scale—an index of difficulty identifying feelings—and the pain vigilance and awareness questionnaire—an index of attention to changes in pain. (**A**) Correlation in all participants. (**B**) Correlation in the Val/Val group. (**C**) Correlation in the Met-carrier group. Red and blue circles indicate the data of Val/Val participants and Met carriers, respectively.

The present study revealed a significant positive correlation between the TAS-20-DIF and PVAQ-ACP scores (Figure 1). This suggests that, similar to the correlation found in patients with chronic pain [10], there exists a correlation between alexithymia and hypervigilance to pain in healthy individuals. In fact, healthy adults with a higher TAS-20-DIF score have a lower tolerance for pain induced by electrical stimulation [11], which supports that individuals with alexithymia are hypervigilant to pain. Furthermore, the correlation between the TAS-20-DIF and PVAQ scores was only significant in the Met-carrier group. In a previous study, a similar significant positive correlation was reported in patients with chronic pain [10]. This suggests that Met carriers, even those who are healthy, may have psychological characteristics that result in a correlation between alexithymia and pain. Some previous studies support the idea that Met carriers have a close correlation between emotion and pain [30,32]. Among patients with fibromyalgia, those with the Met/Met genotype experienced a greater decline in positive affect on days when subjective pain intensity was elevated, compared with those with the Val/Val or Val/Met genotype [32]. Furthermore, patients with the Met/Met genotype reported an increase in pain when pain catastrophizing, which reflects one of the maladaptive emotions to pain, was elevated [30]. Taken together, these studies suggest that COMT polymorphism influences the strength of the correlation between emotional state and pain.

The present study revealed the possible influence of COMT polymorphism on the correlation between alexithymia and hypervigilance to pain. However, the neural substrate underlying this influence remains unknown. A recent systematic review has reported differences in functional brain connectivity between COMT genotypes in several brain regions (e.g., the prefrontal cortex, anterior cingulate cortex, amygdala, and hippocampus) [33]. In terms of functional brain connectivity, Met allele carriers had higher connectivity between the limbic system and prefrontal cortex than individuals with the Val/Val genotype; these areas are closely associated with emotion and pain [34]. These differences in inter-regional connectivity may underlie the genetic influence observed in our study. Further studies are warranted to clarify the neural mechanisms.

The results of the present study should be interpreted in light of some possible limitations. The sample size in this study was relatively small and only young participants were recruited. Future studies with a larger sample size and a wider participant age range are thus necessary to confirm the influence of COMT polymorphism on the correlation between alexithymia and the PVAQ. Furthermore, only one COMT polymorphism (rs4680) was investigated in this study. To understand the influence of other genes involved in the dopamine system, a detailed understanding of dopamine function on the correlation observed in the present study is needed.

In conclusion, in the Met-carrier group, a significant correlation was noted between the PVAQ (an index of excessive attention to pain) and the TAS-20 (an index of alexithymia) subscales. The results of this study suggest that dopamine function influences the correlation between emotional state and pain vigilance. The neural substrates that underlie the observed results should be explored using brain imaging techniques.

## Figures and Tables

**Table 1:** Scores obtained on each subscale of PVAQ and TAS in each COMT group.

	PVAQ-AP	PVAQ-ACP	TAS-20-DIF	TAS-20-DDF	TAS-20-EOT
Val/Val group	23.3 ± 7.1	16.4 ± 4.2	17.7 ± 6.6	15.9 ± 3.9	20.4 ± 3.4
Met-carrier group	24.7 ± 8.1	17.1 ± 6.06	13.7 ± 4.7	14.6 ± 3.4	21.2 ± 3.6
*p* value	*p* = 0.612	*p* = 0.666	*p* = 0.059	*p* = 0.299	*p* = 0.492

Abbreviations: PVAQ-AP: pain vigilance and awareness questionnaire (PVAQ) subscale regarding attention to pain; PVAQ-ACP: PVAQ subscale regarding attention to changes in pain; TAS-20-DIF: the 20-item Toronto Alexithymia Scale (TAS-20) subscale regarding difficulty identifying feelings; TAS-20-DDF: the TAS-20 subscale regarding difficulty describing feelings; TAS-20-EOT: the TAS-20 subscale regarding externally oriented thinking.

**Table 2:** The relationship between each subscale for PVAQ and TAS-20.

		TAS-20-DIF	TAS-20-DDF	TAS-20-EOT
PVAQ-AP	All subjects	r = 0.255 *p* = 0.134	r = 0.145 *p* = 0.40	r = 0.091 *p* = 0.599
	Val/Val group	r = 0.143 *p* = 0.573	r = 0.206 *p* = 0.413	r = 0.278 *p* = 0.264
	Met-carrier group	r = 0.426 *p* = 0.078	r = 0.124 *p* = 0.623	r = −0.080 *p* = 0.753
PVAQ-ACP	All subjects	r = 0.474 *p* = 0.004 *	r = 0.156 *p* = 0.363	r = −0.146 *p* = 0.395
	Val/Val group	r = 0.244 *p* = 0.329	r = 0.158 *p* = 0.530	r = 0.088 *p* = 0.729
	Met-carrier group	r = 0.73 *p* = 0.001 *	r = 0.194 *p* = 0.440	r = −0.316 *p* = 0.201

Abbreviations: PVAQ-AP: pain vigilance and awareness questionnaire (PVAQ) subscale regarding attention to pain; PVAQ-ACP: PVAQ subscale regarding attention to changes in pain; TAS-20-DIF: the 20-item Toronto Alexithymia Scale (TAS-20) subscale regarding difficulty identifying feelings; TAS-20-DDF: TAS-20 subscale regarding difficulty describing feelings; TAS-20-EOT: TAS-20 subscale regarding externally oriented thinking. * *p* < 0.05.

## Data Availability

The data presented in this study are available on request from the corresponding author. The data are not publicly available due to privacy restrictions.
